# Plasmonic Biosensors in Cancer-Associated miRNA Detection

**DOI:** 10.3390/bios15030165

**Published:** 2025-03-04

**Authors:** Nayoung Kim, Mingyu Bae, Euni Cho, Ki Su Kim, Jin-Ho Lee

**Affiliations:** 1 Department of Information Convergence Engineering, Pusan National University, Yangsan 50612, Republic of Korea; skdud0822@pusan.ac.kr (N.K.); mgbae@pusan.ac.kr (M.B.); whdmsdl08@pusan.ac.kr (E.C.); 2 School of Chemical Engineering, College of Engineering, Pusan National University, Busan 46241, Republic of Korea; 3 Department of Organic Material Science & Engineering, College of Engineering, Pusan National University, Busan 46241, Republic of Korea; 4 Institute of Advanced Organic Materials, Pusan National University, Busan 46241, Republic of Korea; 5 School of Biomedical Convergence Engineering, Pusan National University, Yangsan 50612, Republic of Korea; 6 Research Institute of Convergence of Biomedical Science and Technology, Pusan National University, Yangsan 50612, Republic of Korea

**Keywords:** cancer, biosensor, miRNA, surface plasmon resonance, localized surface plasmon resonance, plasmonic enhanced fluorescent

## Abstract

Cancer is one of the most lethal diseases and has distinct variants that affect over 60 organs in the human body. The necessity of advanced methodologies for the early diagnosis of cancer has grown over the past decades. Among various biomarkers, microRNAs (miRNAs) have emerged as highly specific and minimally invasive indicators for cancer detection, prognosis, and treatment monitoring. Their stability in biological fluids and their critical role in gene regulation make them valuable targets for diagnostic applications. Plasmonic biosensors have gained massive attention owing to their unique optical properties, such as surface plasmon resonance, making them promising tools for the sensitive and selective analysis of cancer-associated biomarkers. In contrast to previous reviews, this work offers a comprehensive overview of advancements from approximately the past five years, particularly in the detection of cancer-associated miRNAs. It emphasizes emerging plasmonic sensing strategies, integration with novel nanomaterials, and enhanced signal amplification techniques. By focusing on these recent innovations, this review provides new insights into the potential of plasmonic biosensors to improve cancer diagnosis and treatment.

## 1. Introduction

MicroRNAs (miRNAs), endogenous small non-coding RNA molecules typically consisting of about 20–22 nucleotides, hold a crucial role in cancer development [1,2]. Their post-transcriptional gene regulation ability, such as controlling a gene’s expression by inhibiting protein translation or promoting mRNA degradation, impacts key cellular processes implicated in cancer development and progression, including cell metabolism, proliferation, differentiation, apoptosis, etc. [3,4]. Dysregulation of miRNA expression profiles has been consistently observed in various types of cancer, where specific miRNAs can act as oncogenes or tumor suppressors depending on their target genes [5]. The aberrant expression of miRNAs contributes to the initiation, progression, metastasis, and therapeutic resistance of cancer [6]. As such, miRNAs have emerged as promising biomarkers for cancer diagnosis, prognosis, and therapeutic intervention [7]. Understanding the complicated role of miRNAs in cancer biology offers insights into the underlying molecular mechanisms driving tumorigenesis [8]. It provides opportunities for the development of targeted miRNA-based therapies aimed at treating cancer more effectively.

Precise and accurate detection of miRNAs is challenging due to their short sequence with high similarity, low abundance, and instability [9]. Traditional methods such as northern blotting are widely used to characterize miRNAs but suffer from being slow, semiquantitative, and requiring large samples [10]. Enzyme-based amplification techniques like reverse transcription polymerase chain reaction (RT-PCR) improve sensitivity but are costly and require strict temperature conditions [11,12]. In contrast, enzyme-free approaches like hybridization chain reaction (HCR) and strand displacement amplification (SDA) simplify processes but involve complex probe designs [13]. To this end, as each approach involves trade-offs between sensitivity, complexity, and cost, the development of a precise method of analyzing miRNA holds great promise for understanding cancer mechanisms and developing novel diagnostic and therapeutic strategies.

Owing to the unique optical properties of plasmonic materials, plasmonic biosensors have emerged as a promising platform for the detection and characterization of miRNAs with high sensitivity and specificity [14,15]. Plasmonic biosensors operate on the principle of surface plasmon resonance (SPR), a phenomenon that occurs due to the resonant coupling between electromagnetic waves and the collective oscillations of free electrons present on the surface of plasmonic materials [16,17]. It can be classified into propagating surface plasmon resonance and non-propagating localized surface plasmon resonance (LSPR) according to the transmission mode [18]. These resonance phenomena occur precisely at the interface between dielectric and plasmonic materials, facilitating the alteration and confinement of incident light [19,20]. The size and shape of plasmonic nanoparticles play a crucial role in the performance of these biosensors. As the size of the nanoparticle increases, the LSPR wavelength typically shifts to longer wavelengths (redshift), and the spatial distribution of the electromagnetic field becomes more extensive [21]. Larger nanoparticles can support multiple plasmon modes, leading to more complex field patterns. However, this increase in size also reduces the surface-to-volume ratio, which can diminish the biosensor’s sensitivity to surface-bound analytes. Conversely, smaller nanoparticles exhibit a higher surface-to-volume ratio, enhancing the interaction between the nanoparticle surface and the target molecules. However, if the nanoparticles are too small, quantum confinement effects may become significant, leading to a decrease in the quality factor of the plasmon resonance and reduced electromagnetic field enhancement. Additionally, the shape of the nanoparticles can influence the distribution and enhancement of the electromagnetic field. Nanostructures with sharp edges or corners, such as nanostars or nanorods, can further concentrate the electromagnetic fields, improving the biosensor’s sensitivity. Therefore, optimizing both the size and shape of nanoparticles is crucial for balancing electromagnetic field enhancement with the biosensor’s overall sensitivity and ensuring accurate miRNA detection. These inherent characteristics render plasmonic nanomaterials to detect miRNAs in complex biological samples in a precise and rapid manner, which is crucial for early cancer diagnosis, personalized treatment, and monitoring therapeutic responses [22].

**Figure 1:**
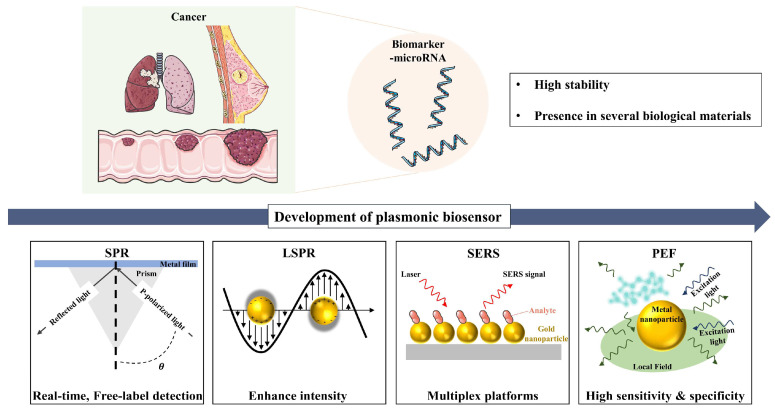
Schematic illustration of plasmonic biosensors for microRNA detection, summarizing key findings and technological progress from SPR to LSPR, PEF, and SERS. Figure created using elements from Servier Medical Art, licensed under CC BY 4.0.

In this review, we aim to provide a comprehensive overview of recent advancements in plasmonic biosensing techniques for cancer-associated miRNA detection, with a particular focus on key methods such as SPR, LSPR, and plasmon-enhanced fluorescence (PEF). This analysis will emphasize the pivotal role of plasmonic nanomaterials in improving biosensor performance, specifically in terms of sensitivity, specificity, and rapid detection. Additionally, we will examine the strengths and limitations of these plasmonic biosensing approaches, as well as the challenges and opportunities for further development. We believe this review will inspire interdisciplinary research, driving significant progress in cancer diagnosis, prognosis, and treatment. See Figure 1.

## 2. Surface Plasmon Resonance-Based Cancer-Associated miRNA Detection

Plasmonic biosensors operate on the principle of surface plasmon resonance (SPR), a phenomenon that occurs when light interacts with the free electrons at the interface between a metal and a dielectric material [23,24]. This interaction excites the electrons, causing them to oscillate collectively [25,26] These collective electron oscillations are known as plasmons, which propagate parallel to the metal surface. The oscillation of plasmons generates an associated electric field that extends approximately 300 nm from the interface between the metal surface and the sample solution. In an SPR sensor, a metal film is illuminated under conditions that excite surface plasmons, leading to a resonance effect highly sensitive to the refractive index of the adjacent medium. When target miRNA molecules bind to complementary probes immobilized on the sensor surface, this binding event increases the local refractive index, causing a measurable shift in the resonance angle or wavelength [16]. This shift is detected in real time, allowing for the quantification of miRNA concentration without labeling. Recent advancements in SPR technology, such as the integration with nanostructures and improvements in sensor chip materials, have further enhanced their analytical performance, making them highly effective for miRNA detection. The binding of miRNAs to the sensor surface induces changes in the local refractive index, leading to measurable shifts in the SPR signal [27]. This shift can be quantified to provide real-time information about the presence, concentration, and binding kinetics of miRNAs [28].

In this regard, Mujica et al. harnessed the SPR phenomenon to develop a label-free genosensor for microRNA-21 quantification [29]. The platform of the genosensor was designed to form bilayers of poly (diallyldimethylammonium chloride) (PDDA) and graphene oxide (GO) on a mercapto propane sulfonate (MPS)-modified gold disk by self-assembly procedure. The amine-modified DNA probe facilitates the covalent attachment on the sensor surface via the carboxyl group opening of GO. The analytical performance of the biosensors was evaluated by testing the influence of the number of PDDA/GO bilayers on the sensor response. Accordingly, the proposed platform exhibited femtomolar quantification of microRNA-21 in a label-free manner, with a linear range between 1.0 fM and 10 nM and a detection limit of 0.3 fM. The utilization of advanced materials and innovative design approaches can significantly enhance the performance of SPR-based biosensors.

Building on these advancements, Xue and his group have utilized a different 2D nanomaterial, antimonene, and developed a label-free surface plasmon resonance sensor for miRNA-21 and miRNA-155 [30]. Their density functional theory (DFT) calculations exhibit stronger chemical interactions between antimonene and both single-stranded DNA (ssDNA) and double-stranded DNA (dsDNA) compared to graphene. Theoretically, this enhanced interaction results from the greater delocalization of the 5s/5p orbitals in antimonene. Based on this theoretical finding, they developed an SPR-based sensor that could reach attomolar-level quantification of clinically relevant biomarkers such as miRNA-21 and miRNA-155. In more depth, Singh et al. theoretically investigated the utilization of antimonene on a gold (Au)-coated BK-7 prism for SPR-based biosensors, demonstrating better sensitivity than conventional and Au-graphene-based SPR biosensors [31]. The unique properties of antimonene, such as its stability, high adsorption energy, and strong immobilization capability for miRNA biomarkers, can be leveraged to enhance the performance of the proposed antimonene-based SPR biosensor, which is essential for enhancing SPR biosensor efficiency. This enhanced sensitivity is achieved through the superior immobilization of target molecules (miRNA) facilitated by antimonene on the surface of the SPR biosensor, which is essential for enhancing SPR biosensor efficiency.

**Figure 2:**
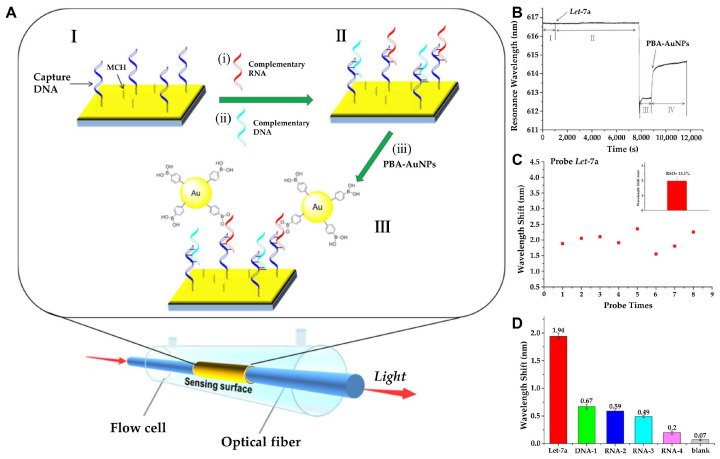
(**A**) Schematic representation of miRNA detection by fiber-optic SPR sensing system: (**I**) Functionalization of the sensing surface with capture DNA and 6-Mercapto-1-hexanol (MCH). (**i**,**ii**) The capture DNA binds with the target RNA with a specific sequence through complementary pairing. (**II**) Single-stranded RNA or DNA hybrid on the sensing surface. (**iii**) The plasmonic AuNPs and propagating plasmons on the gold surface can further induce signal amplification in the SPR sensing system. (**III**) PBA-AuNPs selectively bind with RNA. (**B**) Real-time sensorgram depicting the detection of Let-7a (10⁻^8^ M) using the fiber-optic SPR sensor: (**I**) Introduction of hybridization buffer. (**II**) Injection of Let-7a in hybridization buffer. (**III**) Rinsing with water. (**IV**) Introduction of PBA-AuNPs dispersed in water. (**C**) Signal stability analysis of PBA-AuNP-mediated amplification for Let-7a (10⁻^8^ M). (**D**) Evaluation of the selectivity of the miRNA SPR sensor: detection of Let-7a (perfectly complementary target), DNA-1 (perfectly complementary sequence), RNA-2 (single-nucleotide mismatch), RNA-3 (single-nucleotide deletion), RNA-4 (random sequence), and blank control (absence of RNA or DNA). All nucleic acids were tested at a concentration of 10⁻^8^ M. (**A**–**D**): Reproduced with permission from [32], published by ACS Sensors 2018.

Although the detection of small analytes is still challenging for SPR-based biosensors, as these analytes cause slight refractive index changes, Qian et al. employed phenylboronic acid functionalized gold nanoparticles (PBA-AuNPs) as extrinsic labels to amplify the signal in a fiber optic-based SPR sensing system (Figure 2) [32]. Through these PBA-AuNPs, target miRNA can be selectively recognized, as boronic acid can specifically bind with cis-diol groups, as it lacks in DNA. The result shows that PBA-AuNPs can selectively amplify the signal of target miRNA (Let-7a), with a detection limit of 0.27 pM. Similarly, Li et al. utilized two layers of GO-AuNPs composites as both the sensing substrate and the signal amplification element to develop a sensitive SPR biosensor [33]. GO-AuNPs were functionalized with a Au film surface and thiolated DNA was covalently adhered to the AuNPs as a capture probe. The sandwich structure was formed in the presence of target miRNA while introducing the DNA functionalized GO-AuNP composite detection probe, which resulted from the DNA/RNA hybridization. In their explanation, both layers of GO-AuNPs localized on the top and bottom of the sandwich structure were used to amplify the SPR response. The surface plasmon of AuNPs might be resonantly excited and create a strong local electromagnetic field, which, combined with the high mass density and dielectric constant of AuNPs, enhances the SPR response. The electromagnetic coupling between the AuNPs on the upper layer of GO-AuNP composites and the underlying Au film could also amplify the SPR response. In addition, a higher surface area of the GO-AuNPs at the bottom layer could also promote more structural formation of DNA-GO-AuNPs on the sensor surface to enhance the SPR response. As a result, a clear response could be observed in the case of target miRNA-141 (1 pM), either alone or with other interfering molecules, such as miRNA-200a and miRNA-429. Furthermore, owing to the high performance of the sensor system, the relative expression levels of target miRNA-141 in three different human cancer cell lines [i.e., prostate carcinoma cell lines (22Rv1), hepatocellular carcinoma cell lines (SMMC-7721), and colon cancer cell lines (LoVo)] were able to be distinguished as well.

While many researchers focused on signal amplification, in another approach, Nie et al. utilized DNA tetrahedron probes (DTPs) on gold surfaces to create a low-fouling surface, minimizing nonspecific adsorption from complex sample matrices while specifically capturing the target molecule, let-7a [34]. In parallel, the SPR sensor successfully detected 2 pM let-7a, exhibiting noticeable signal degradation over seven cycles. The standard deviation was approximately 1% (n = 7), indicating the excellent reproducibility of the DTP–Au chip. In the presence of let-7a, a sandwich structure formed between the DTPs, the target let-7a, and DNA-linked AuNPs through hybridization. This structure enhanced the SPR signal due to electronic coupling between the surface plasmon of the gold film and the localized plasmon of the AuNPs. Additionally, the AuNPs were further enlarged using a catalytic growth procedure, amplifying the electronic coupling effect while preventing random growth on the gold film due to the antifouling properties of the DTPs. Taking these advantages, the SPR sensor exhibited high sensitivity toward let-7a, detecting levels as low as 0.8 fM, and was capable of detecting miRNA in undiluted human serum and cancer cell lysates. The advancements in plasmonic biosensors, particularly through the incorporation of advanced materials such as nanomaterials and innovative designs, have significantly enhanced the sensitivity, specificity, and applicability of SPR sensors for the detection of miRNAs.

As discussed above, SPR biosensors are highly sensitive to environmental changes, including temperature fluctuations, pH variations, and ionic strength [35]. These factors can alter the refractive index near the sensor surface, leading to signal drift and reduced reproducibility. Implementing precise environmental control systems within the biosensor setup can mitigate the effects of external fluctuations. Maintaining constant temperature, pH, and ionic strength conditions ensures consistent sensor performance and enhances reproducibility. Another major challenge in SPR biosensors is non-specific adsorption, which can interfere with device functionality, potentially hindering the accurate detection of biological targets present at low concentrations [36]. The immobilization of biorecognition elements, such as DNA probes or antibodies, onto the sensor surface, is critical for specific miRNA detection. Over time, these immobilized molecules can degrade or detach, leading to decreased sensor performance and reproducibility issues. Additionally, surface fouling and non-specific interactions further compromise sensor stability. To address these challenges, advanced surface modification methods have been developed, including the use of self-assembled monolayers and polymer coatings, which minimize non-specific adsorption and enhance the durability of the sensor surface. By integrating innovative surface functionalization techniques and environmental control strategies, researchers continue to improve the stability and reproducibility of SPR biosensors for clinical applications in miRNA-based cancer detection. Moreover, incorporating machine learning (ML) algorithms to analyze SPR signal variations presents a promising approach to further enhance biosensor accuracy. The recent research on surface plasmon resonance-based cancer-associated miRNA detection is compared in Table 1.

## 3. Localized Surface Plasmon Resonance-Based Cancer-Associated miRNA Detection

Localized Surface Plasmon Resonance (LSPR) biosensors have emerged as a promising tool for the sensitive and specific detection of biomolecules, including miRNAs, due to their ability to measure refractive index changes induced by biomolecular interactions [37,38]. LSPR-based sensors typically require simpler optical setups compared to SPR [39]. The absence of a need for prism coupling in LSPR allows for more compact and cost-effective instrumentation, which is also less susceptible to mechanical vibrations and temperature fluctuations [40,41]. This simplicity not only reduces the overall cost but also enhances the robustness and user-friendliness of the sensors. Unlike SPR sensors, which rely on the interaction of light with free electrons at planar metal–dielectric interfaces, LSPR sensors utilize localized plasmon oscillations on the surface of metallic nanostructures [42]. These oscillations result in enhanced optical absorption and scattering, particularly for nanostructures larger than a few tens of nanometers, and are strongly influenced by the size, shape, and composition of the nanostructures [43]. LSPR exhibits a decay length of the electromagnetic field on the order of 6 nm, with both SPR and LSPR demonstrating exponential decay. The shorter decay length of LSPR minimizes sensitivity to fluctuations in the refractive index of the surrounding solution, while simultaneously enhancing sensitivity to changes in the refractive index at the surface [44,45]. The high sensitivity of LSPR peak shifts to changes in the local refractive index makes it a powerful tool for detecting small molecules, such as miRNAs, in various applications.

Based on these principles, Zhang et al. developed a label-free, ultrasensitive nanoprobe based on LSPR for the detection of miR-205, a biomarker associated with lung cancer [46]. The sensor utilized single DNA-modified gold nanocubes (AuNCs), which exhibit enhanced sensitivity due to their square structure and multiple vertices, inducing a broader range of surface plasmon resonances. During hybridization of the target miRNA with thiolated single-stranded DNA (ssDNA) immobilized on the AuNCs, shifts in the LSPR scattering spectrum were detected in real time, providing quantitative measurements of miRNA hybridization. The sensor achieved a detection range of 10 pM to 1 μM and a low detection limit of 5 pM in serum samples. Furthermore, the probe demonstrated high specificity, effectively discriminating between perfectly matched, single-base mismatched, and random RNA sequences. Notably, the AuNC probes demonstrated superior detection efficiency and sensing stability compared to Au-nanosphere-based probes, underscoring the structural advantages of the nanocube design. Expanding on the structural considerations for LSPR sensitivity, Portela et al. employed nanogap antennas for the detection of miRNA-210, a relevant biomarker for lung cancer diagnosis [47]. Utilizing a simple and low-cost hole-mask colloidal lithography technique, they fabricated large-area sensor chips (cm^2^-sized) with uniform plasmonic responses. These chips featured gold nanodisks separated by gaps averaging 11.6 ± 4.7 nm, demonstrating a bulk refractive index sensitivity of 422 nm per RIU, validated through Finite-Difference Time-Domain (FDTD) numerical simulations. The biosensing capabilities of these sensor chips were evaluated using DNA and miRNA hybridization to detect miRNA-210, achieving a limit of detection (LOD) of 0.78 nM (5.1 ng/mL) without additional amplifications. Through their research, they also confirm that smaller gap sizes enhance sensitivity as a result of improved electromagnetic field conditions. Additionally, the optical characterization revealed that parallel orientations of the nanogap antennas offer higher sensitivity than perpendicular orientations.

**Figure 3:**
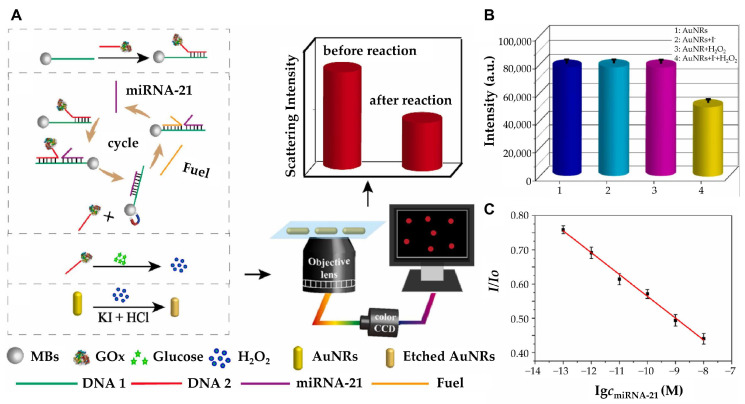
(**A**) Schematic diagram of high-sensitivity detection of microRNA-21 with strand displacement amplification strategy under DFM. (**B**) The scattering intensity of AuNRs under different conditions. 1: AuNRs; 2: AuNRs +1 mM I^−^; 3: AuNRs +1 μM H_2_O_2_; 4: AuNRs +1 mM I^−^ + 1 μM H_2_O_2_. (**C**) The linear plot of the scattering intensity ratio changes versus the miRNA-21 concentration. (**A**–**C**): Reproduced with permission from [48], published by Biosensors and Bioelectronics 2022.

While these approaches focus on optimizing nanostructure geometry to enhance LSPR sensitivity, an alternative strategy involves chemically modulating the plasmonic properties of nanostructures for improved detection performance. In this regard, Zhang et al. developed a highly sensitive and specific platform for miRNA-21 detection using I_2_-triggered chemical etching of AuNRs under dark-field microscopy (Figure 3) [48]. Upon target miRNA-21 hybridization, a strand displacement amplification (SDA) reaction triggers enzymatic cleavage, releasing iodine ions that selectively etch AuNRs. This process leads to a significant blue shift and intensity decrease in the LSPR scattering, providing a highly sensitive optical readout. With a dynamic detection range of 0.1–10,000 pM and a low detection limit of 71.22 fM (3σ/k) via dark-field microscopy, the platform demonstrated exceptional specificity, distinguishing single-base mismatches while maintaining high recovery rates in human serum samples. Beyond chemical modulation, DNA-programmed nanostructures offer a highly efficient approach for multiplexed and ultrasensitive miRNA detection. Wu and his group developed a biosensor that integrates DNA-programmed Au-on-Ag heterostructure and DNA tetrahedral frameworks (DTFs) to develop a multiplex and ultrasensitive detection of exosomal miRNAs associated with non-small cell lung cancer (NSCLC) [49]. DTF improves probe binding efficiency by reducing entanglement, enhancing mechanical stability, and providing antifouling properties. The biosensor captures exosomal miRNAs with DTF probes immobilized on a gold array chip. Then, Ag nanocubes (AgNCs) functionalized with ssDNA hybridize with the miRNAs. Gold nanoparticles (AuNPs) assemble on the AgNC surface to form the Au-on-Ag heterostructure, generating amplified SPR responses. Through this synergy, a broad detection range from 2 fM to 20 nM with an ultra-low detection limit of 1.68 fM was achieved.

To further push the sensitivity of LSPR biosensors, Al Mubarak et al. developed a plasmonic nucleotide hybridization chip capable of detecting miRNA-155 at concentrations as low as 80 aM [50]. The chip utilizes 1,4-benzenedithiol self-assembled monolayers (SAM) to immobilize AuNPs on the substrate. A hairpin DNA capture probe chemisorbed onto the AuNPs binds miRNA-155 via specific G–C base pairing. Subsequent hybridization with Fe_3_O_4_@AuNPs carrying detection probes completes the double hybridization mechanism, further amplifying the surface plasmon signal. This design bridges localized Au nanoparticles (AuNPs) on a gold substrate with core/shell Fe_3_O_4_@Au nanoparticles (NPs) carrying detection probes. The amplification is driven by the plasmonic resonance between the Au NPs and the gold shell of the core/shell NPs, alongside the scattering band of the core/shell material resonating with an 800 nm light source. The combination of these two nanoparticle components produces highly consistent and reproducible intensity signals, confirming the robustness of the biosensor across different chips. As a result, this system not only achieves exceptional sensitivity through the synergistic effects of localized plasmon resonance and resonant scattering but also demonstrates excellent reproducibility, making it highly suitable for ultra-low analyte concentration detection. On the other hand, Miti et al. explored an alternative signal amplification strategy by combining LSPR with a hybridization chain reaction (HCR) to develop a biosensor for the specific and sensitive detection of miRNA-17 [51]. The biosensor utilized surface-immobilized AuNPs functionalized with highly specific hairpin probes for miRNA recognition. Signal amplification was achieved through HCR, an isothermal and enzyme-free method that enhanced the LSPR response. This system achieved a detection limit of approximately 1 pM within a rapid analysis time of less than 1 h, demonstrating its efficiency and practicality for clinical diagnostics. Expanding on these efforts to enhance sensitivity while ensuring specificity, Liyanage et al. introduced a tapered optical fiber (TOF) plasmonic biosensor incorporating gold triangular nanoprisms (AuTNPs) [52]. This system not only enhanced plasmonic sensitivity but also addressed nonspecific binding by integrating poly (ethylene glycol)-thiol (PEG-SH) as an antifouling spacer. As a result, the biosensor successfully distinguished cancerous from noncancerous samples with a *p*-value < 0.0001 and achieved ultra-low detection limits ranging from 179 to 580 aM.

**Figure 4:**
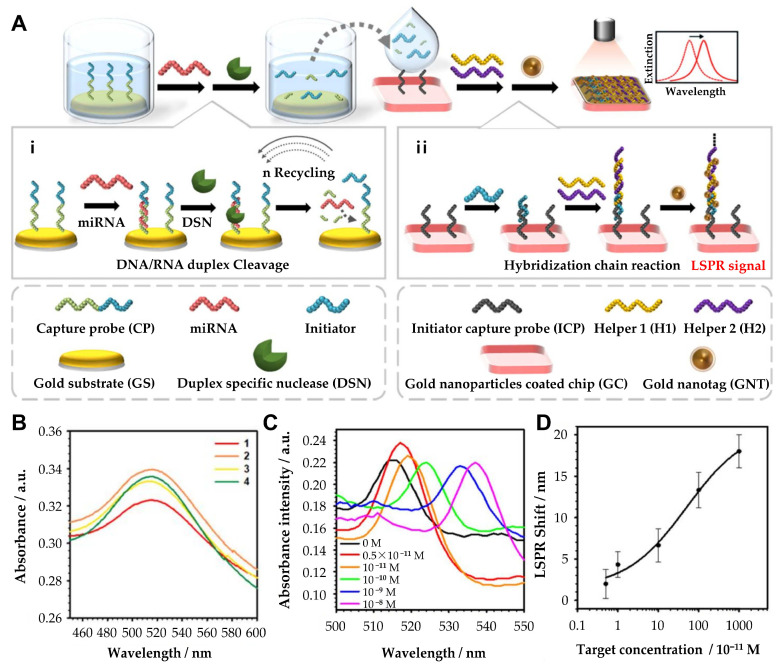
(**A**) Principle of the hybrid signal amplification for LSPR-based miRNA sensing. (**i**) Duplex-specific nuclease-assisted target miRNA recycling and generation of intermediate ssDNA, initiator probe (IP). (**ii**) Initiator probe-induced formation of the DNA sandwich assemblies. (**B**) LSPR spectra at each reaction step. (1) Gold nanoparticle-coated chip (GC); (2) initiator capture probe-conjugated GC (GC/ICP); (3) initiator probe-conjugated GC + ICP (GC/ICP/IP); and (4) DNA self-assembly with H1 and H2 on GC + ICP + IP (GC/ICP/IP/H1/H2). (**C**) Absorbance spectra of the HCR-tGNT-based miRNA sensors toward miR-10b. (**D**) Determination of the limit of detection with various concentrations (5 × 10^−11^, 10^−11^, 10^−10^, 10^−9^, and 10^−8^ M). (**A**–**D**): Reproduced with permission from [53], published by ACS Applied Materials & Interfaces 2019.

In addition, researchers have also focused on developing portable and label-free LSPR biosensors for practical applications. Ki et al. developed a portable, label-free LSPR biosensor for the sensitive and selective detection of miRNA-10b, a biomarker associated with gastric cancer (Figure 4) [53]. The system integrated an enzyme-assisted target recycling mechanism with tannic acid-capped AuNPs to achieve high sensitivity. The detection process involves duplex-specific nuclease (DSN)-assisted target recycling, which produces intermediate DNA fragments that trigger the formation of super-sandwich self-assemblies. These assemblies facilitate the adsorption of tannic acid-capped AuNPs via hydrogen bonding, generating a detectable LSPR signal. The system demonstrates a ratiometric readout with intrinsic self-calibration, correlating miRNA levels to changes in the absorption spectrum. The biosensor achieves a detection range of 5 pM to 10 nM and a detection limit of 2.45 pM. This portable biosensor correlated miRNA-10b expression with tumor size, invasion depth, metastasis, and prognosis, showcasing its potential for clinical applications. The continuous advancements in nanostructure design, signal amplification techniques, and multiplexing capabilities have significantly enhanced the performance of LSPR-based miRNA biosensors. The recent research on localized surface plasmon resonance-based cancer-associated miRNA detection is compared in Table 2.

## 4. Surface Enhanced Raman Scattering-Based Cancer-Associated miRNA Detection

While localized surface plasmon resonance (LSPR)-based plasmonic biosensors have demonstrated exceptional sensitivity in miRNA detection, another widely utilized plasmonic sensing technique, Surface Enhanced Raman Scattering (SERS), offers complementary advantages, particularly in the detection of low-abundance biomarkers. Given its remarkable sensitivity and molecular specificity, SERS has been extensively applied in cancer-associated miRNA detection. SERS is a technique that amplifies the Raman scattering signal of molecules adsorbed on a nanostructured metal surface [55]. The signal amplification primarily occurs through electromagnetic and chemical mechanisms. The electromagnetic mechanism involves the generation of a strong electric field due to LSPR on the metal nanostructure surface, which enhances the Raman scattering signal of the molecule. The chemical mechanism arises from the modification of the molecule’s polarizability as a result of physicochemical interactions with the metal surface. SERS has been widely applied for detecting low-concentration molecules, including DNA [56,57], microRNA [58], proteins [59,60,61], and bacteria [62,63]. It has also been used for single-cell [64,65] detection and identification, bioimaging, and disease diagnosis, providing detailed structural information about biological analytes.

One notable strategy for enhancing SERS sensitivity involves the use of advanced nanomaterials. For instance, Liu et al. developed a SERS-based sensing strategy using a three-component MXene/MoS2@AuNPs (MMA) system for ultrasensitive detection of miRNA-182, a key biomarker directly implicated in cancer development [66]. In this system, 2D materials such as MXene interact with analyte molecules via hydrogen bonding, coordination interactions, and van der Waals forces, stabilizing Raman peaks for quantitative detection. Simultaneously, MoS2 generated high SERS response signals to its nanostructure and high electron transfer performance, and gold nanoparticles (AuNPs) act as hotspots, further amplifying the SERS signal and adsorbing hairpin probe DNA via Au-S bonding. In the presence of miRNA-182, the hairpin probe DNA reacted and released Cyanine 5 (Cy5) molecules, which decreased the intensity of the SERS peak at 1362 cm^−1^. The MMA-based synergistic SERS strategy exhibited a linear detection range of 10 aM to 1 nM for miRNA-182 and achieved a limit of detection (LOD) value of 6.61 aM, which was evaluated.

In a different approach, Guerrini et al. employed gold-coated paramagnetic nanoparticles to capture SERS-active AuNPs, thereby creating an enhanced plasmonic hot-spot environment for miRNA biomarker detection [67]. This sandwich-type assay approach allows the specific and selective identification of miRNAs, such as miR-141, through an amplified Raman scattering signal. The incorporation of paramagnetic nanoparticles not only facilitates the isolation and enrichment of target miRNAs from complex biological fluids but also improves both detection sensitivity and specificity. The study demonstrated a detection limit in the attomolar range, highlighting the capability of SERS biosensors to detect miRNAs at clinically relevant concentrations. Similarly, Treerattrakoon et al. developed a magnetic separation-based SERS platform for miRNA-29a detection [68]. The SERS tags are composed of a DNA detection probe on the AuNR surface through a self-assembly process with 4-mercaptobenzoic acid (4-MBA), and a DNA-conjugated magnetic nanoparticle (MNP) is used as the capture probe to develop a hybridization and sandwich complex-based SERS platform. The SERS tags were created by attaching the Raman-active molecule 4-MBA onto AuNRs and conjugating complementary reporter DNA sequences to the target miRNA. The target miRNA was captured by MNPs conjugated with the capture DNA probe, and hybridization led to SERS target labeling. A sandwich complex of MNP/miRNA/SERS tag is formed, and the assembled SERS tag generates multiple hotspots that enhance the SERS signal due to the presence of the target miRNA. In the absence of the target miRNA, the hybridization complex could not be formed and the SERS tag was washed away during the washing process after magnetic separation, resulting in no detectable SERS signal. When a sample containing 1 μM of miRNA-29a was analyzed via SERS, a strong characteristic spectrum of 4-MBA was recorded compared to the negative control without miRNA-29a. Further evaluation using various concentrations of miRNA-29a (0–1000 pM) demonstrated that the SERS signal intensity decreased linearly with decreasing concentration, with LOD of 10 pM.

To meet the clinical need for simultaneous detection of multiple miRNAs, multiplexed SERS sensor arrays have been developed. Si et al. developed a catalytic hairpin assembly (CHA)-based SERS sensor array for multiplex miRNA detection [69]. This sensor formed a SERS sensor array chip with four detection units by immobilizing one of four different hairpin structure DNA sequences on each of four Au/Ag alloy nanoparticle (AuAgNP)-coated detection wells to simultaneously measure multiple miRNAs in one sample. Interactions between the AuAgNP layer of the wells and AuAgNP-based SERS tags generated hot spots, enhancing Raman signals and improving detection sensitivity. The Raman signal intensity gradually increased with increasing miRNA concentration, exhibiting a good linear relationship (R^2^ = 0.98) from 0.50 pM to 100 nM. Even in the presence of three other miRNAs at a relatively high concentration (1.0 nM), no significant changes were observed in the Raman response, demonstrating excellent specificity.

**Figure 5:**
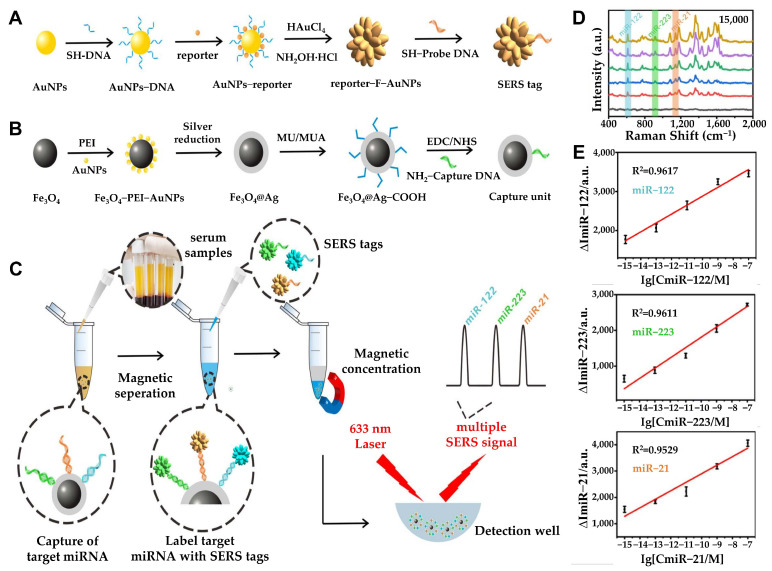
(**A**) Schematic processes of synthesizing SERS tag. (**B**) Design and synthesis of the capture substrate. (**C**) Schematic illustration of multiplex miRNA assay via the SERS sandwich strategy. Detection procedure of multiple miRNAs based on the capture substrate/miRNA/SERS tag sandwich structure. (**D**) SERS spectra for triple-target miRNA detection (blank, 1 fM, 100 fM, 10 pM, 1 nM, and 100 nM). (**E**) Standard curves of the SERS peak intensity at 615, 918, and 1140 cm^−1^ against the concentrations of miR-122 (**top**), miR-223 (**middle**), and miR-21 (**bottom**), respectively. The error bars stand for the standard deviation of triplicate measurements. (**A**–**E**): Reproduced with permission from [70], published by ACS Sensors 2021.

This platform combines the signal amplification capability of CHA reactions and the strong SERS enhancement of AuAgNPs, making it a promising tool for multiplex miRNA detection. Similarly, Wu et al. developed a SERS-based magnetic-assisted sandwich biosensor for the multiplex detection of three hepatocellular carcinoma (HCC)-associated miRNA biomarkers (miRNA-122, miRNA-223, and miRNA-21) in serum [70] (Figure 5). This sensor uses Ag-coated magnetic nanoparticles (AgMNPs) and star-shaped fractal gold nanoparticles (F-AuNPs), and the F-AUNPs and AgMNPs are immobilized with three probe DNAs, and the three capture DNAs specifically recognize the three target miRNAs to form sandwich hybridization complexes. F-AuNP has the same core–shell structure as nanobridge nanogap particles (Au-RNNPs), which have been used in recent years, but many more nanogaps appeared on the rough surface of F-AuNPs. Therefore, F-AuNPs formed many electromagnetic hot spots, which greatly enhanced the SERS intensity, leading to its advancement. In addition, AgMNPs are composed of Fe3O4 cores coated with Ag, which simplifies the separation step and improves reproducibility, and they show excellent SERS properties and are used as excellent capture substrates due to their magnetic response and low nonspecific adsorption. As a result of measuring the SERS spectra in human serum, the LODs of three miRNAs (miRNA-122, miRNA-223, and miRNA-21) were measured as 349 aM for miRNA-122, 374 aM for miRNA-223, and 311 aM for miRNA-21. From a clinical perspective, label-free approaches can simplify the detection process by eliminating the need for target labeling, thereby reducing complexity and potential interference. For example, Crawford et al. developed a label-free, homogeneous SERS-based inverse molecular sentinel (iMS) nanobiosensor for cancer diagnosis, enabling the early detection of esophageal adenocarcinoma (EAC) and other diseases without the need for target amplification [71]. The detection mechanism of this sensor’s iMS is based on hairpin-shaped DNA strands that undergo structural changes in the presence of a specific biomarker. The distance-dependent nature of the SERS signal is utilized as a transduction mechanism by utilizing plasmonic-active silver-coated gold nanostars (AuNS@Ag). The SERS iMS nanoprobe detects “OFF-to-ON” signals based on a nonenzymatic strand displacement process and structural change of the stem-loop (hairpin) oligonucleotide probe upon target binding. In the absence of miRNA-21, the iMS system exhibits a low SERS spectral signal due to the placeholder strand maintaining a stable linear duplex structure, keeping Cy5 far from the nanostar’s plasmonic surface. Conversely, when miRNA-21 is present, it binds to the toehold region of the probe, inducing a hairpin structural change that brings Cy5 closer to the plasmonic surface of the nanostar, thereby enhancing the SERS signal due to increased electromagnetic field interactions. The iMS nanobiosensor demonstrated LOD of 4.6 pM for miRNA-21, confirming its high sensitivity. Since the iMS homogeneous assay does not require target labeling or additional washing steps, it represents a versatile and powerful tool applicable across a wide range of diagnostic applications.

**Figure 6:**
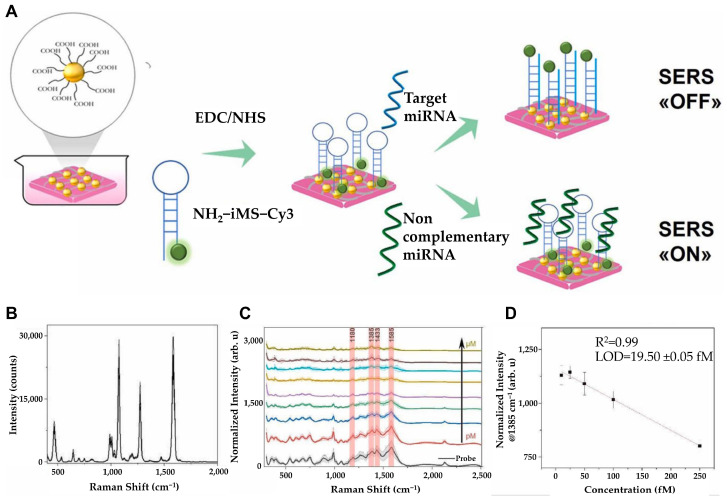
(**A**) Schematics for the detection strategy by designed substrate. (**B**) Average BPT SERS spectrum. (**C**) SERS spectrum of nanofiber composites incubated with only iMS DNA probe (black) or with target miRNA at 20 μM (dark yellow), 10 μM (brown), 5 μM (cyan), 1 μM (orange), 500 μM (purple), 100 μM (green), 1 μM (blue), 100 μM (red). Characteristic peaks of Cy3 are marked as red at 1180 cm^−1^, 1385 cm^−1^, 1433 cm^−1^, and 1585 cm^−1^. (**D**) Linear range of the curve between 10 and 250 fM target miRNA concentrations. (**A**–**D**): Reproduced with permission from [72], published by Elsevier 2025.

Building on this label-free concept, Martino et al. developed a 3D flexible biosensor incorporating nanofibers (NFs), AuNPs, and iMS technology for the early detection of miRNA-223-3p, a novel biomarker for laryngeal cancer, which has a high mortality rate due to late diagnosis [72] (Figure 6). The iMS DNA probe was immobilized on AuNPs for selective detection of miRNA-223-3p. The probe was functionalized with an amino group (-NH_2_) on one end, while the other end was labeled with Cyanine 3 (Cy3) as a Raman reporter molecule. The stem-and-loop structure of iMS changes its conformation upon interaction with miRNA-223-3p. In the absence of miRNA 223-3p, NH_2_-iMS-Cy3 remains in a closed state, positioning Cy3 near the nanoparticle to generate a strong SERS signal. However, in the presence of the target miRNA, the loop region hybridized with miRNA-223-3p, inducing a structural change that caused Cy3 to move away from the nanoparticle surface, resulting in a significant decrease in the SERS signal. This mechanism demonstrated the probe’s high specificity for the target miRNA. Using various concentrations of miRNA-223-3p diluted in salt buffer (SB), the sensor exhibited a linear detection range of 10–250 fM and a LOD of 19.50 ± 0.05 fM. This sensor does not require amplification and offers key advantages including sensitivity and flexibility.

Alternatively, hybrid platforms that integrate SERS with electrochemical detection have also been developed to further enhance the sensitivity of SERS-based biosensors. Wang et al. introduced an innovative SERS–electrochemical hybrid biosensor designed for the highly sensitive detection of miRNA-141, a crucial biomarker implicated in various cancers [73]. The Au@Ag nanowires not only provide strong plasmonic enhancement for Raman signal amplification but also contribute to electrochemical signal transduction. The combined SERS–electrochemical approach enables the detection of miRNA-141 at sub-femtomolar concentrations while improving detection accuracy and minimizing false positives. Such hybrid platforms demonstrate the potential of multimodal biosensors for early-stage cancer diagnostics.

SERS-based biosensors are significantly influenced by factors such as the signal and stability of SERS nanotags [74]. However, in actual analysis, reproducibility issues may arise due to the characteristic that peak positions shift depending on the thickness of the molecular layer on the nanoparticle surface, as seen in the dispersion of single-point spectra. This means that signal intensity may vary even under identical conditions, leading to reduced reliability and making it challenging to maintain consistent biosensor performance. Given the need to address these challenges, one possible approach could be the use of the multiphoton effect in experiments, which may enable the identification of various cancer cells through nonlinear multiphoton interactions. This method enhances the detection of biological agents as a non-invasive tool by providing more comprehensive information and improving sensitivity. In addition, artificial intelligence (AI), machine learning (ML), and soft computing can deliver improved results in multiple research areas by providing agility and efficiency across a variety of applications. ML is related to the ability of machines to infer approximate solutions from past data or discover patterns and rules in unknown data. This suggests that ML holds great potential for enhancing efficiency and precision in data analysis. Therefore, by applying ML and soft computing to address the signal fluctuation and reproducibility issues of SERS-based biosensors, sensor data patterns can be learned and optimized, ultimately improving signal reliability and sensitivity. This advancement enables more precise biomarker detection and real-time analysis, which is expected to accelerate the development of next-generation biosensor technology. The recent research on surface plasmon resonance-based cancer-associated miRNA detection is compared in Table 3.

## 5. Plasmon-Enhanced Fluorescent-Based Cancer-Associated miRNA Detection

Beyond SPR-based detection, plasmon-enhanced fluorescence (PEF) has recently emerged as a powerful strategy for miRNA sensing [81]. PEF amplifies fluorescence signals by modulating the interaction between fluorophores and localized surface plasmons, resulting in increased signal intensity, extended fluorescence lifetime, and reduced photobleaching [82]. This enhancement is achieved by precisely positioning fluorophores near plasmonic nanostructures—such as gold (Au) or silver (Ag) nanoparticles—where localized electromagnetic field enhancement significantly boosts fluorescence emission [83,84]. Compared to conventional fluorescence-based detection techniques, PEF-based miRNA biosensors offer higher sensitivity and improved signal-to-noise ratios, making them particularly advantageous for detecting low-abundance miRNAs in complex biological environments [85]. PEF offers significant potential in biosensing, but achieving optimum, reproducible signal enhancement, while precisely controlling the dye-to-metal distance, still remains a key challenge [84]. To address these limitations, researchers have explored diverse strategies, each contributing uniquely to advancing the field.

Building on the concept of assembly-based fluorescence enhancement, one promising development achieved by Peng et al. is the development of a highly sensitive nanogap antenna-based fluorescent sensor for miRNA-21 detection and imaging [86]. This system utilizes the target-triggered end-to-end (ETE) assembly of Au NRs to enhance fluorescence through the PEF effect at the gap region by integrating primary fluorescence amplification through the PEF effect and secondary amplification via strand displacement amplification (SDA) reaction. In the presence of miRNA-21, a target-triggered ETE assembly of Au NRs forms, positioning fluorophores in a gap region where fluorescence transits from quenching to enhancement. Through this dual-amplification strategy, combined with single-molecule counting, ultra-sensitive miRNA detection with a detection limit of 97.2 aM was achieved. The approach also enabled single-molecule counting and distinguished different cell types based on miRNA expression levels, highlighting its potential for precise miRNA profiling. Further advancing the multiplexing and sensitivity of miRNA detection, Qu et al. also employed the nanoparticle assembly-based platform that utilizes DNA-bridged AuNR and upconverting nanoparticle (UCNP) assemblies [87]. By forming a core–satellite geometry, this platform enabled luminescence resonance energy transfer (LRET) for efficient fluorescence emission, significantly reducing signal cross-talk and achieving high signal-to-noise ratios. With limits of detection (LODs) of 3.2 zmol/ngRNA for miR-21 and 10.3 zmol/ngRNA for miR-200b, the platform allows for multiplexed detection and supports the simultaneous quantification of these biomarkers in both in vitro and in vivo conditions. As the upconversion fluorescence from UCNPs minimizes spectral overlap with dyes like TAMRA and Cy5.5, quantitative imaging of miRNA markers in living cells and animal models could be obtained, offering a versatile tool for epigenetics research and personalized medicine. In another approach, Hwang et al. developed DNA-engineered ultraflat-faceted core–shell nanocuboids (FANCs) to provide strong, quantitative PEF signals for sensitive miRNA detection [88]. These nanocuboids were engineered by functionalizing DNA strands with fluorophores at one end and thiols at the other, which were densely modified onto AuNRs before the growth of ultraflat Ag nanoshells. This design enabled precise control of fluorophore placement relative to the Ag surface, resulting in a fluorescence enhancement factor of ~186. Additionally, fluorescent silica shell-coated FANCs (FS-FANCs) were subsequently synthesized to stabilize fluorescence and improve signal intensity. Embedding multiple fluorophores in the silica shell further minimized signal fluctuation due to positional changes in the solution. Computational modeling confirmed that the strongest fluorescence enhancement occurs at vertex and vertex-adjacent regions of the FANC structures, consistent with experimental findings. This design was applied in microarray assays for miRNA detection, achieving a dynamic range from 100 aM to 1 pM, and demonstrated over 1000-fold greater sensitivity than traditional chemical fluorophores, overcoming the limitations of traditional microarrays, such as small sample sizes and lack of target amplification. By overcoming the limitations of conventional microarrays, such as small sample sizes, low sensitivity, and lack of target amplification, FS-FANCs offer a highly robust and stable platform for ultrasensitive biosensing applications. 

**Figure 7:**
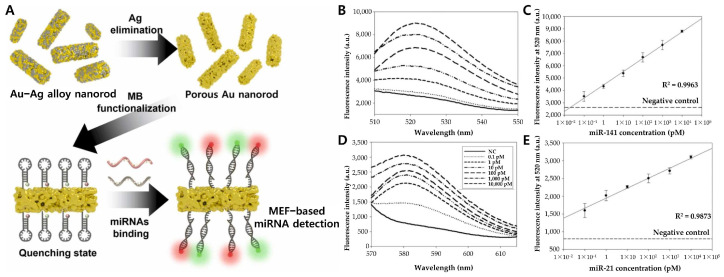
(**A**) Schematic diagram of MEF-based multi-miRNAs nanobiosensor. (**B**) Fluorescent spectrum and (**C**) linear curve of the MB-functionalized porous Au nanorod with target miRNA (miR-141) from 0.1 pM to 10,000 pM. (**D**) Fluorescent spectrum and (**E**) linear curve of the MB-functionalized porous Au nanorod with target miRNA (miR-21) from 0.1 pM to 10,000 pM. (**A**–**E**): Reproduced with permission from [89], published by Sensors and Actuators B: Chemical 2023.

Further expanding the potential of PEF-based biosensors, Choi et al. utilized a porous gold nanorod (PAuNR) platform for the simultaneous detection of multiple miRNAs via the PEF effect, such as miR-141 and miR-21 (Figure 7) [89]. PAuNRs were synthesized via electrochemical deposition of gold and silver, followed by selective etching of Ag to create a porous structure. Unlike traditional Au nanomaterials, PAuNRs amplified fluorescence across the visible spectrum, facilitating simultaneous multi-target detection. Molecular beacons (MBs) specific to miR-141 and miR-21 were tagged with FAM and TAMRA, respectively, and immobilized on the PAuNRs. In the absence of target miRNAs, fluorescence was quenched by proximity to the Au surface; however, upon hybridization with complementary targets, fluorescence signals were restored and amplified via the PEF effect, allowing multiplexed detection with enhanced sensitivity that yielded ultra-low detection limits of 0.1 pM.

Similarly, in an effort to expand the PEF phenomenon across fluorescent materials with various emission wavelengths, Liu et al. made advancements in near-infrared (NIR)-driven biosensors, demonstrating exceptional potential for sensitive miRNA detection in clinical samples [90]. This biosensor utilizes polydopamine-coated upconversion nanoparticles (UP/Au) for PEF-based fluorescence enhancement in combination with a two-step toehold-mediated strand displacement (TMSD) amplification strategy. First, when miRNA-155 binds to single-stranded DNA (ssDNA)-1 on wrinkled silica nanoparticles (WSNs), it forms a complex and exposes a new toehold. In the next step, ssDNA-2 on the UP/Au surface interacts with ssDNA-1, displacing miRNA-155 and forming an ssDNA-1/ssDNA-2 complex. This cyclic amplification increases the fluorescence signal, achieving a detection range of 0.5–20 pM and a limit of detection as low as 19.76 fM. Using NIR-excited upconversion nanoparticles reduced background noise and false positives, while polydopamine enhances biocompatibility and fluorescence performance via PEF. The WSNs provide abundant reaction sites for probe immobilization, while the enzyme-free detection process minimizes errors. Consequently, it demonstrates high sensitivity and practical applicability in serum sample analysis.

**Figure 8:**
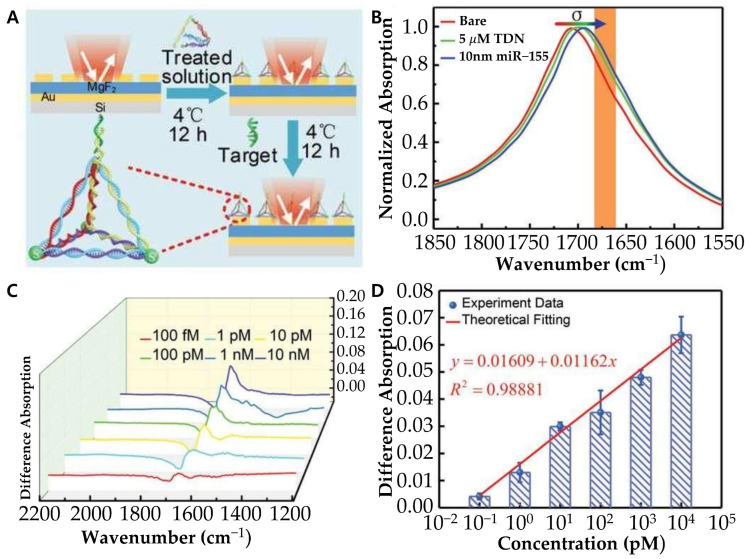
(**A**) Schematic diagram of experimental preparation procedure via tetrahedral DNA nanostructure (TDN). (**B**) Measured normalized absorption spectra of the bare MPA (red), and the MPA after physisorption of TDN (green) and subsequent binding with miR-155 with 10 × 10^−9^ m concentration (blue). (**C**) Difference absorption spectroscopy of different concentrations of miR-155. (**D**) Differential signal as a function of miR-155 concentrations. (**A**–**D**): Reproduced with permission [91], published by Advanced Science 2021.

Expanding on this, Hui and colleagues developed a surface-enhanced infrared absorption (SEIRA)-based biosensor that integrates tetrahedral DNA nanostructures (TDNs) with a metal–insulator–metal perfect absorber (MPA) to enhance infrared absorption, providing an alternative yet highly sensitive approach for miRNA-155 detection (Figure 8) [91]. The TDN consists of four ssDNA strands with three thiol-modified ends anchored onto the platform surface, while the fourth strand serves as a probe for miRNA-155 capture. The tetrahedral structure (~6 nm thick) ensures precise probe orientation, creating a solution-like reaction environment that improves binding efficiency and sensor stability. To further enhance detection sensitivity, a metal–insulator–MPA composed of metallic nanoantennas, a dielectric spacer, and a gold sheet was chosen as the plasmonic platform due to its strong infrared absorption and near-field enhancement capabilities. When miRNA-155 binds to the TDNs, its molecular vibrations interact with the MPA, producing a distinct spectral peak. Maximum signal amplification occurs when the miRNA-155 vibration frequency aligns with the MPA’s resonance frequency. This biosensor demonstrated a 1000-fold improvement in sensitivity, achieving LOD as low as 100 fM, approximately 5000 times more sensitive than ssDNA probes, and 100 times more sensitive than fluorescence-based methods. To further enhance the performance of plasmonic biosensors, in another aspect, Schmidt and colleagues further refined detection strategies by integrating rolling circle amplification (RCA) to enhance sensitivity for detecting low concentrations of target analytes [92]. Their key innovation was the use of a biointerface with guiding ssDNA strands, which confined RCA-generated ssDNA chains within the evanescent surface plasmon field, optimizing sensor response. By controlling the conformation and spatial organization of these ssDNA chains, they significantly improved detection efficiency. The study revealed that adjusting the surface density of anchored ssDNA enhances sensor sensitivity, improving the LOD by approximately two orders of magnitude compared to direct labeling. Furthermore, fluorescence microscopy enabled the visualization of individual binding events, allowing for single-molecule detection and further advancing biosensor performance. Importantly, their approach demonstrated excellent reproducibility, with fluorescence signal variations systematically quantified, achieving a chip-to-chip reproducibility of ~10%, ensuring consistent and reliable sensor performance.

Building on the advancements in PEF-based biosensors, plasmon-enhanced electrochemiluminescence (ECL) sensors have emerged as another powerful approach for improving miRNA detection sensitivity. Feng et al. developed an innovative ECL biosensor that utilizes DNA-templated Ag nanoclusters as ECL emitters and gold nanoparticles (AuNPs) as the LSPR source for detecting miRNA-21 [93]. In this system, a novel enzyme-free catalytic hairpin assembly (CHA) strategy was applied. In detail, two DNA hairpins (H1 and H2) were designed to undergo a CHA reaction. When miRNA-21 is present, it binds to H1 and opens its structure. H1 then hybridizes with H2 to form an H1–H2 duplex, releasing miRNA-21, which triggers multiple cycles of amplification and generates H1–H2 duplexes. These duplexes are immobilized on an AuNP-modified electrode via Au–S bonding. Ag nanoclusters are then grown on the duplexes, generating a strong ECL signal for miRNA-21 detection. Through a CHA amplification strategy, the system demonstrates a wide linear detection range (from 1 aM to 104 fM) with a detection limit of 0.96 aM for miRNA-21. Similarly, Lu et al. developed a highly sensitive biosensor using gold inverse opals (GIOs) as plasmonic nanostructures and Ru(bpy)_3_^2^⁺-doped silica nanoparticles (RuSi NPs) as ECL emitters [94]. The interaction between the gold surface and RuSi NPs creates a strong electromagnetic (EM) field, significantly boosting the ECL signal. By optimizing surface morphology, the sensor achieved a detection limit of 3.3 fM for miRNA-21.

**Figure 9:**
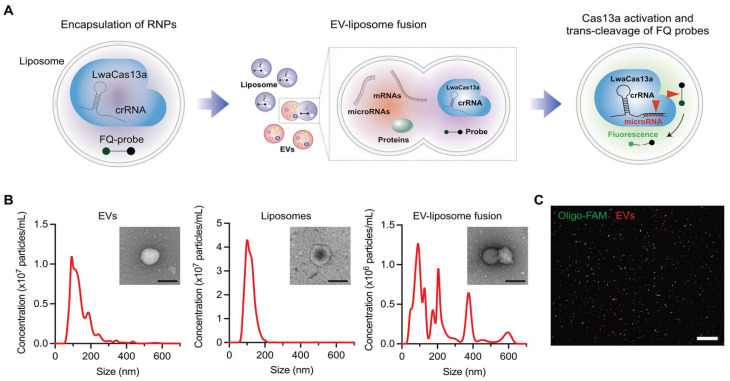
(**A**) Schematic diagram for the CRISPR/Cas13a-based EV miRNA detection assay. (**B**) Size distributions of EVs (**left**), liposomes (**middle**), and liposome–EV fusion products (**right**). The insets show transmission electron micrographs (TEMs) of an EV, liposome, and fusion particle. Scale bar, 200 nm. (**C**) Representative fluorescence image of EV–liposome fusion products: fluorescently labeled EVs (red) and liposomes containing FAM-labeled oligos (green). Scale bar, 100 µm. (**A**–**C**): Reproduced with permission from [96], published by Advanced Science 2023.

Expanding on plasmon-enhanced ECL strategies, recent advancements have extended this approach to exosomal miRNAs. In this aspect, Peilin Wang et al. developed a plasmonic nanocavity-modulated ECL sensor specifically designed for detecting exosomal miRNA-223-3p, a biomarker associated with gastric cancer [95]. By integrating circular Au nanoplate-film, AuNPs, and tetrahedral DNA nanostructures, the sensor enhances the local density of optical states (LDOS), significantly amplifying the spontaneous ECL signal from PEDOT-modified S dots. The AuNPs function as nanoantennas, and by optimizing nanocavity parameters, the system effectively localizes the electromagnetic field, generating high-density hotspots that facilitate high-efficiency electron injection into the ECL emitters. This sensor demonstrates a broad dynamic range (1 fM to 1 nM) and a low detection limit (0.14 fM) for miRNA-223-3p detection in ascitic fluid. Another notable breakthrough study in the detection of miRNAs in extracellular vesicles (EVs) focused on a CRISPR/Cas13a-based sensing system. Jeong et al. encapsulated the CRISPR components (Cas13a, crRNA, and FQ probes) in liposomes, which can fuse with EVs, enabling the detection of miRNA targets without the need for RNA extraction or amplification [96]. By counting miR-21-5p-positive EVs in cancer patient plasma, this system achieved a limit of quantification of 0.14% and a high fusion efficiency (>80%) for delivering sensing materials into EVs (Figure 9).

Furthermore, researchers have extended this approach to single-cell analysis. Liu et al. developed a plasmon-enhanced droplet screening platform for high-throughput miRNA quantification at the single-cell level [97]. This platform integrates microfluidic droplet-based single-cell encapsulation with plasmonic nanosensors, enabling precise detection and quantification of intracellular miRNAs. The platform employs two distinct types of Ag nanoparticle (AgNP)-based nanosensors: (1) capture nanosensors, which are functionalized with nucleic acid sequences complementary to the target miRNA, and (2) indicator nanosensors, which are conjugated with a fluorophore-labeled oligonucleotide. In the absence of target miRNAs, these nanosensors form sensor–sensor complexes via plasmonic coupling, producing strong fluorescence. However, when target miRNAs are present, they competitively hybridize with the capture nanosensors, disrupting the sensor–sensor complexes and causing a corresponding reduction in fluorescence intensity. This fluorescence modulation enables highly sensitive miRNA detection with a detection limit of ~0.1 nM. Single-cell miRNA concentrations are quantified by measuring fluorescence signals within individual droplets, allowing high-throughput analysis. The platform processes approximately 1000 droplets per second using dark-field microscopy, enabling statistical analysis of ~100 single cells per experiment. This system was validated in MCF-7 breast cancer cells, by successfully distinguishing miR-155 in the cytoplasm and miR-25 in the nucleus, demonstrating its capability for subcellular miRNA localization. This platform provides an advanced tool for profiling miRNA heterogeneity at the single-cell level. Its ability to resolve nucleocytoplasmic miRNA distributions offers valuable insights into intracellular RNA dynamics, with potential applications in cancer diagnostics, biomarker discovery, and single-cell transcriptomics.

PEF-based biosensors have demonstrated significant potential in detecting miRNAs due to their heightened sensitivity and specificity. However, achieving optimal signal enhancement in PEF-based biosensors presents several challenges. A primary challenge is the precise control over the distance between fluorophores and metal nanostructures [98]. The fluorescence enhancement is highly dependent on this spacing; deviation can lead to quenching rather than signal enhancement [99]. Maintaining an optimal gap is crucial for maximizing the electromagnetic field enhancement while preventing non-radiative energy transfer that causes quenching. Another significant issue is the reproducibility of nanostructure fabrication [100]. Variations in the size, shape, and arrangement of metal nanoparticles can result in inconsistent plasmonic properties, leading to variability in sensor performance [101]. Achieving uniformity in nanostructure fabrication is essential for reliable and reproducible biosensor responses. Addressing these issues through innovative fabrication and stabilization strategies will be pivotal in advancing the practical application of PEF-based biosensors in biomedical diagnostics. The recent research on plasmon-enhanced fluorescent-based cancer-associated miRNA detection is compared in Table 4.

## 6. Conclusions and Future Perspectives

The growing recognition of miRNAs as crucial biomarkers in cancer has significantly influenced the development of diagnostic and treatment strategies. Given their role in essential cellular processes like tumorigenesis, metastasis, and drug resistance, miRNAs have become invaluable for early cancer detection and monitoring. Among the various detection platforms, plasmonic biosensors have emerged as one of the most promising technologies due to their exceptional sensitivity, rapid detection capabilities, and ease of integration into clinical approaches. This review highlights the recent advancements in plasmonic biosensing techniques, particularly SPR, LSPR, and PEF. These techniques utilize the unique optical properties of plasmonic materials to enhance detection sensitivity and specificity, overcoming the limitations of traditional diagnostic platforms.

However, despite the significant advancements in plasmonic biosensors for miRNA detection, several challenges still hinder their full clinical potential. First, reproducibility and stability are critical issues that must be addressed. Variations in sensor fabrication, surface functionalization, and environmental factors can introduce variability, which impacts reliability and consistency. Second, while plasmonic biosensors have made considerable progress in sensitivity, detecting low-abundance miRNAs still remains a significant challenge. Achieving detection limits that are compatible with clinical needs, especially for early-stage cancer detection, requires further optimization of the biosensor platform. Additionally, advancements in nanomaterial design and optimizing the interaction between the target miRNAs and the plasmonic nanomaterials, while minimizing non-specific binding and interference, are other key hurdles to overcome as well. Furthermore, the development of multiplexed systems capable of detecting multiple miRNAs or biomarkers will offer a more comprehensive analysis of cancer profiles. Plasmonic biosensors are rapidly closing the gap with conventional diagnostic technologies due to advances in sensor design, nanomaterial integration, surface functionalization, and multiplexing technologies, and they show great potential in clinical practice where personalized healthcare and real-time high-precision diagnosis are required. This could lead to more accurate diagnosis and personalized treatment strategies.

Nevertheless, several challenges must be addressed before these technologies can be widely adopted in clinical practice. While the COVID-19 pandemic has accelerated routine clinical sample analysis and improved sample accessibility, the lack of automated platforms, the complexity of system operation, and regulatory hurdles continue to impede the widespread adoption of plasmonic biosensors. In particular, clinical validation and regulatory approval processes require significant time and investment. A clearer discussion of specific regulatory procedures and ongoing research efforts is necessary to bridge this gap. Addressing key issues such as non-specific binding and developing multiplexed detection systems will be crucial for the successful clinical application of plasmonic biosensors in early cancer detection and personalized treatment strategies.

In conclusion, while challenges still remain in enhancing reproducibility, sensitivity, and specificity, plasmonic biosensors offer great promise for advancing cancer diagnostics and treatment. Notably, the incorporation of multiplexing technologies enables precise control of small samples, increasing throughput and facilitating real-time detection, which in turn allows for more accurate treatment adjustments. Furthermore, their combination with microfluidic and lab-on-a-chip systems could drive the development of automated diagnostic platforms. These advancements will accelerate the development of next-generation biosensor technology, bringing us closer to highly efficient, real-time, and personalized cancer diagnostics.

## Figures and Tables

**Table 1:** Comparison of linear ranges and detection limits on surface plasmon resonance-based cancer-associated miRNA detection.

Materials	Cancer Type	Biomarker	Linear Range	LOD	Reference
MPS, PDDA, GO	cervical cancer, prostate cancer	miRNA-21	1.0 fM~10 nM	0.3 fM	[29]
Antimonene, Graphene, AuNR	-	miRNA-21, miRNA-155	-	10 aM	[30]
PBA, AuNPs, MCH	-	Let-7a	1 pM~100 nM	0.27 pM	[32]
GO, AuNPs	-	miRNA-141, miRNA-200	0.1 pM~2.0 nM	0.1 fM	[33]
DTP, AuNPs	breast cancer, lung cancer, hepatocellular carcinoma	Let-7a	0.8 fM~2 pM	0.8 fM	[34]

**Table 2:** Comparison of linear ranges and detection limits on localized surface plasmon resonance-based cancer-associated miRNA detection.

Materials	Cancer Type	Biomarker	Linear Range	LOD	Reference
a single DNA-modified gold nanocube	Lung cancer	miR-205	10 pM~1 μM	5 pM	[46]
nanogap antennas		miRNA-210	-	0.78 nM	[47]
gold nanorods	-	miRNA-21	0.1~10,000 pM	71.22 fM	[48]
DNA tetrahedral frameworks, silver nanocube	Non-small cell lung cancer	miRNA-21, miRNA-378, miRNA-200, miRNA-139	2 fM~20 nM	1.68 fM	[49]
magnetic/gold core/shell nanoparticles	-	miRNA-155	0.08~10 fM	80 aM	[50]
gold nanoparticles	-	miR-17	-	1 pM or 50 amol	[51]
gold triangular nanoprisms	Prostate cancer	let-7a, let-7c, miR-200b, miR-141, and miR-21	-	179 and 580 aM	[52]
tannic acid-capped gold nanoparticles	-	miR-10b	5 pM~10 nM	2.45 pM	[53]
tapered optical fibers, gold nanoparticles, gold nanorods, gold triangular nanoprisms	-	Let 7a, Let 7c, miRNA 141, miRNA 21, miRNA 200	1 fM~100 nM	103 aM and 261 aM	[54]

**Table 3:** Comparison of linear ranges and detection limits on surface-enhanced Raman scattering resonance-based cancer-associated miRNA detection.

Materials	Cancer Type	Biomarker	Linear Range	LOD	Reference
Fe3O4, Ag, Au, dithiobis-(2-nitrobenzoic acid) (DTNB)	Pancreatic cancer	miRNA-10b	3 aM~100 pM	1 aM	[75]
4-MBA, AuNRs, MNP	-	miRNA-29a	0~1000 pM	10 pM	[68]
Mxene, MoS2, AuNP	-	miRNA-182	10 aM~1 nM	6.61 aM	[66]
AuAgNP, 4-mercaptobenzonitrile (MPBN)	-	miRNA-21, miRNA-1246, miRNA-221, miRNA-133a	0.5 pM~100 nM	0.15 pM	[69]
AuNP, NFs, iMS DNA	Laryngeal cancer	miRNA-223-3p	10~250 fM	19.50 ± 0.05 fM	[72]
AgMNPs, F-AuNPs	Liver cancer	miRNA-122, miRNA-223, miRNA-21	1 fM~10 nM	miRNA-21: 311 aM, miRNA-122: 349 aM, miRNA-223: 374 aM	[70]
AgNPs	breast cancer	human breast cancer-associated miRNAs (e.g., let-7b, miRNA-1, miRNA-10b, miRNA-125b, miRNA-126, miRNA-133a, miRNA-143, miRNA-155 and miRNA-21)	-	~ =1 aM	[76]
Fe3O4 Nanoparticles (Fe3O4 NP), Au/Ag core-shell nanorods	breast cancer	miRNA-21, miRNA-155, let 7b	-	miRNA-21: 0.05 fM, miRNA-155: 0.063 fM, let 7b: 0.037 fM	[77]
iMS probe, bimetallic nanostars (BNS)	colorectal cancer	miRNA-21, miRNA-221	-	miRNA-21: 6.8 zmol, miRNA-221: 16.7 zmol	[78]
AgNR, AuNP, functionalized multiple-armed tetrahedral DNA nanostructure (FMTDN)	lung cancer	miRNA-21, miRNA-486	-	-	[79]
Au nanoarrays (Au NAs)	liver cancer	miRNA-224	1 fM~1 nM	0.34 fM	[80]
Ag-coated AuNS, iMS probe	esophageal cancer	miRNA-21	0~1 nM	4.6 pM	[71]

**Table 4:** Comparison of linear ranges and detection limits for plasmon-enhanced fluorescent-based cancer-associated miRNA detection.

Materials	Cancer Type	Biomarker	Linear Range	LOD	Reference
AuNR, Cy5	-	miRNA-21	0.1~1.0 pM	97.2 aM	[86]
AuNR, TAMRA, Cy5.5	-	miRNA-21, miR-200b	5~1000 pM (miRNA-21) 10~500 pM (miR-200b)	0.11 aM (miRNA-21)/0.34 aM (miR-200b)	[87]
AuNR, AuNPs, Ag	-	miRNA-134	100 aM~1 pM	1.0 fM	[88]
AuNR, Ag, MB, TAMRA	-	miRNA-21, miRNA141	0.1 pM~10 nM	51.1 fM (miRNA-141)/30.6 fM (miRNA-21)	[89]
WSN, ssDNA1, ssDNA2, AuNPs, PDA	Cervical Cancer	miRNA-155	0.5~20 pM	19.76 fM	[90]
MPA, TDN	-	miRNA-155	-	0.1 pM	[91]
Au, oligonucleotide (RCA)	-	ssDNA	-	13 pM	[92]
DNA-AgNC, AuNPs, MCH	-	miRNA-21	1 aM~10 pM	0.96 aM	[93]
GIO, RuSi NP	-	miRNA-21	5.0 fM~5.0 pM	3.3 fM	[94]
AuNPs, TDN, PEDOT conducting polymer	Gastric Cancer	miRNA-223-3p	0.065 pM~0.65 nM	4.0 pM	[95]
Gold nanowell	Cholangiocarcinoma	Tumor-derived extracellular vesicles (tEVs)	-	7.5 × 10^7^ EVs	[96]
AgNP	-	miRNA-155, miRNA-25	0.1 nM~1000 nM	0.1 nM	[97]
